# Myomodulation with Injectable Fillers: An Innovative Approach to Addressing Facial Muscle Movement

**DOI:** 10.1007/s00266-018-1116-z

**Published:** 2018-03-16

**Authors:** Maurício de Maio

**Affiliations:** Clinica Dr. Maurício de Maio, Avenida Ibirapuera, 2907 cj 1202, São Paulo, SP Moema EP: 04029200 Brazil

**Keywords:** Myomodulation, Injectable fillers, Hyaluronic acid, Esthetic facial procedures, Palsy

## Abstract

**Abstract:**

Consideration of facial muscle dynamics is underappreciated among clinicians who provide injectable filler treatment. Injectable fillers are customarily used to fill static wrinkles, folds, and localized areas of volume loss, whereas neuromodulators are used to address excessive muscle movement. However, a more comprehensive understanding of the role of muscle function in facial appearance, taking into account biomechanical concepts such as the balance of activity among synergistic and antagonistic muscle groups, is critical to restoring facial appearance to that of a typical youthful individual with facial esthetic treatments. Failure to fully understand the effects of loss of support (due to aging or congenital structural deficiency) on muscle stability and interaction can result in inadequate or inappropriate treatment, producing an unnatural appearance. This article outlines these concepts to provide an innovative framework for an understanding of the role of muscle movement on facial appearance and presents cases that illustrate how modulation of muscle movement with injectable fillers can address structural deficiencies, rebalance abnormal muscle activity, and restore facial appearance.

**Level of Evidence V:**

This journal requires that authors assign a level of evidence to each article. For a full description of these Evidence-Based Medicine ratings, please refer to the Table of Contents or the online Instructions to Authors www.springer.com/00266.

## Introduction

Theories of facial aging have largely focused on changes in skin, underlying fat, and bone that result in sagging and folds [1], while the role of muscle in aging has generally been neglected [2]. The complementary and distinct ways in which injectable fillers and neuromodulators have generally been used for rejuvenation and improvement of facial esthetics [3] illustrate how skin and fat are considered separately from muscle action. Injectable fillers are customarily used to fill static wrinkles, folds, and localized areas of volume loss [4–7]. Neuromodulators (such as onabotulinumtoxinA) are used to reduce muscle movement in overacting muscles, for example, to diminish hyperdynamic lines or correct position or asymmetry by reducing muscle activity [8–13]. However, long-term observations of patients with certain structural deficiencies treated only with injectable fillers suggest that fillers can also be used to alter muscle movement (myomodulation) in facial esthetic treatments and may provide another tool, in addition to neurotoxins, in the armamentarium of facial muscle modulation.

In the facial literature, consideration of the role of functional muscle groups, including synergists and agonist/antagonist pairs, is focused on the opposing actions of brow levators and depressors, which has guided clinical practice [14, 15]. However, it is clear that functional muscle groups contribute to facial movement and appearance, not just in the example of the brow, but throughout the face. Structural deficiency in either bone or fat pads can precipitate abnormal movement patterns, and such an imbalance, whether resulting from congenital structural deficiencies or changes in muscle action with aging, is reflected in both movement and appearance. The aim of this article is to introduce the concept of using injectable filler treatment to modulate facial muscle action and to present cases that illustrate the use of this approach. Filler treatment can be used to address muscle imbalance that results from structural defects with or without volume loss, and therefore, cases without substantial volume loss were selected to illustrate this approach.

## Understanding Muscle Movement in the Face

Mimetic muscles have several characteristics that differentiate them from skeletal muscles. Generally, mimetic muscles have their origin in bone and insert on the skin and among the fibers of other muscles, with no tendons, except for the sphincteric muscles [16]. In addition, mimetic muscles appear to lack typical muscle spindles [17, 18], which function in resetting resting tone. Together with these characteristics, three key aspects underlie the role of muscle movement in facial expression: length–tension relationship, muscle pulley and lever systems, and the action of functional muscle groups.

### Length–Tension Relationship

The force that a muscle produces is described in part by the length–tension relationship, which relates to two components in a muscle model: a contractile component (active tension, produced by contraction of the muscle) and an elastic component (passive tension, resulting from the elasticity of associated tendon and connective tissues). Peak force is produced by the contractile component of the muscle at resting length and is reduced if the muscle fiber is either shortened or stretched. Passive tension increases with increasing length in the elastic component: As the connective tissue associated with the muscle is pulled, it resists and pulls back when released.

For mimetic muscles that have no tendons and insert in the skin [16], the elasticity of the skin and connective tissue is the primary source of the elastic component of the length–tension relationship. Loss of skin elasticity in aging thus alters the length–tension relationship for mimetic muscles: The muscle’s ability to return to rest after contraction and to maintain the resting position of the skin is diminished. Moreover, loss of elasticity, together with the decrease in fat compartment and bone bulk, leads to sagging of the skin [19], which further stretches the facial muscle in a domino effect.

### Muscle Pulley and Lever Systems

Biomechanical fixed pulley systems alter the angle of action of muscles, and levers increase their mechanical advantage, enhancing muscle force or displacement [20, 21]. An example of such a biomechanical system in the body is the patella [20]. The lateral suborbicularis oculi fat pad (SOOF), located at the lateral/inferior orbital rim and deep to the orbicularis oculi and zygomaticus major [22, 23], acts as a pulley glide plane [1] and, together with bone, as a lever fulcrum for the zygomaticus major muscle. Acting over the SOOF appears to provide a mechanical advantage to the zygomaticus major, which lifts the corners of the mouth in a smile. In aging, the loss of structure beneath the muscle, either from loss of bone or the loss and/or ptosis of fat, might decrease that fulcrum effect, reducing the muscle’s force and diminishing its ability to lift the corner of the mouth.

### Functional Muscle Groups

Groups of muscles (agonists and antagonists) working in harmony contribute to a normal, youthful appearance in facial expression. Levators and depressors work in opposition, and their interactions underlie facial appearance at rest and in dynamic expression. In youth, levators are usually stronger than depressors [24, 25]. The levator muscle’s ability to maintain the position of soft tissue structures of the face is counteracted by the downward pull of gravity and the pull of its depressor antagonists. However, the balance between antagonist muscles may be disrupted, due to structural deficiencies in youth or due to bone and/or soft tissue loss in aging: If a levator muscle lacks or loses lifting power, the depressor is freed to act with reduced opposition.

Consider the synergistic levators of the upper lip: In youth, the zygomaticus major and minor muscles play a critical role in making the corner of the mouth tilt up in a smile. If the zygomaticus major has reduced lifting power due to a lack of underlying structural support, the relative role of the risorius muscle increases and produces a more horizontal smile. If zygomaticus major lifting capacity is further diminished, the depressor anguli oris (DAO) muscle predominates, and a “DAO smile,” with the corners of the mouth downturned, is observed [11, 26]. The lack of underlying structure leading to DAO smile may result from changes over time in aging, or it may occur in youth due to structural deficiency.

## Addressing Muscle Movement with Injectable Fillers

**Fig. 1:**
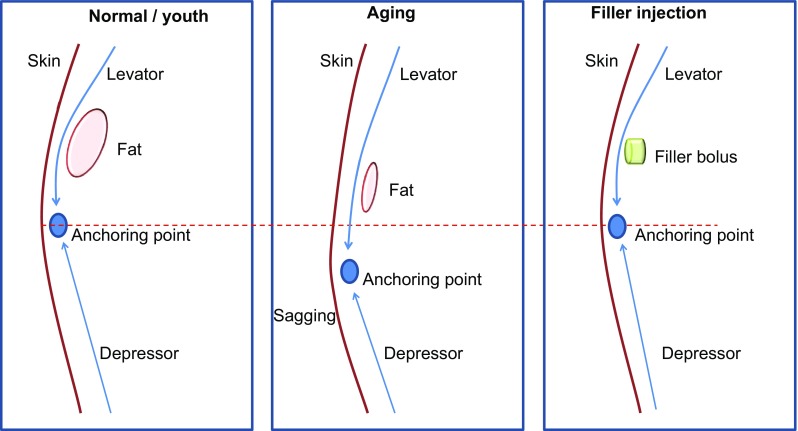
Aging and hypothetical treatment effects on muscle action. Normal youthful facial structure (underlying bone and fat) gives muscle fibers convexity, which allows powerful contraction of levator muscles. The levator and depressor muscles are balanced, and the structures are in their normal/youth position. In aging, underlying support for muscle is lost with deflation of facial structure. I believe that the mechanical advantage provided by the lever fulcrum is diminished and the levator loses lifting power to counteract gravity. Muscle fibers are stretched as skin sags. With reduced opposition, the depressor increases in tone over time and pulls facial structures downward in a domino effect. A filler bolus replaces lost structure (fulcrum), increasing mechanical advantage of the levator muscle. The levator’s movement is facilitated. This reduces sagging and balances contraction of the depressor, halting the chain of events triggered by aging

Each of these biomechanical concepts contributes to our understanding of changes in muscle movement during aging, as well as in cases of facial palsy. In the typical youthful face, there is a clear convexity of the upper cheek due to intact zygomatic arch, malar fat, and SOOF. Under these ideal conditions there is optimal pulling force of the zygomaticus major muscle. In aging, midface volume loss, displacement of fat pads, and loss of skin elasticity may alter zygomaticus major muscle action. The distance between the zygomaticus major origin in the zygomatic bone and insertion in the modiolus area at the corner of the mouth [27] increases when skin sags due to deflation of fat pads, and as a result, the corner of the mouth falls (Fig. 1). The stretching of the fibers of the zygomaticus major results in a loss of resting tension and power in contraction. At the same time, any mechanical advantage of the lever effect over the lateral SOOF is reduced as SOOF volume is depleted. Consequently, the zygomaticus major can no longer adequately counterbalance the downward gravitational pull and the contracting force of the DAO [11, 26].

**Fig. 2:**
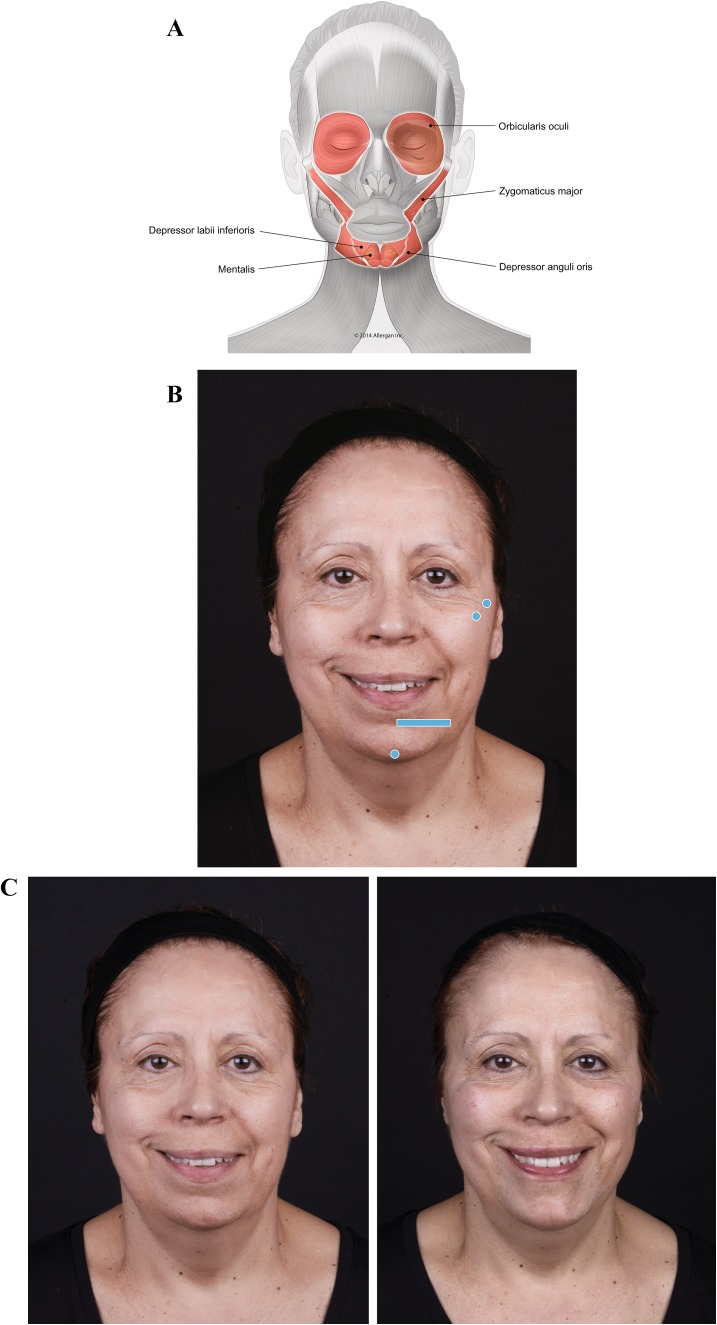
Case 1: Asymmetric smile. The patient was treated on both sides at the zygomatic arch and on the chin on her left side only. Voluma was injected at the bone, 0.1 mL at the zygomatic arch on her right side and 0.1 mL at the zygomatic arch and 0.1 mL at the zygomatic eminence on her left, using a 27-g needle to increase the mechanical advantage of the zygomaticus major. Voluma was injected in labiomental angle superficial to the depressor anguli oris (DAO) (0.7 mL) and chin apex (0.3 mL) using a 25-g blunt microcannula and a fanning technique. **a** Muscles involved. **b** Injection sites (blue markings; dot = bolus injection, bar = fanning). **c** Before treatment (left), the patient has a stronger action of zygomaticus major on her right side (zygomatic smile). On her left side, we notice that her zygomatic is weaker and her DAO is stronger compared with her right side, creating a DAO smile. Note that immediately after treatment (right), the corner of her mouth on her left side is lifted and her smile is more symmetrical. The corners of her mouth are more evenly positioned due to facilitation of zygomaticus major muscle bilaterally and reduced contraction of the DAO on the patient’s left side

Case 1, a mature woman with an asymmetrical smile, illustrates some of these effects (Fig. 2). On her right side, she presents a zygomatic smile, but on her left she shows a DAO pattern (Fig. 2c, left). Before treatment, her left cheek sags, yielding a more prominent nasolabial fold. The zygomaticus major is less efficient on her left side, and the balance between weakened zygomaticus major and its antagonist, DAO, has been lost. DAO is now free to pull down the corner of the mouth.

The presence of fillers can act mechanically to alter muscle movement by either facilitating their action, via a lever or pulley effect, or decreasing contracting by blocking their movement (Table 1). A bolus of filler injected under (deep to) a muscle increases its convexity, acting as a fulcrum to increase mechanical advantage. Conversely, injecting filler more superficially may reduce contraction by impeding muscle movement. These are simple mechanical effects.

**Table 1:** Addressing muscle movement with filler injection, theoretical basis

	Decreased muscle movement	Increased muscle power
Underlying mechanism	Increased distance between origin and insertion stretches muscle fibers and decreases muscle movement	Shortened distance between origin and insertion increases movement in contraction
Possible causes	Loss of convexity due to lack of fat or bone support	Deficit in underlying bony structure or shortened bone structure
Examples	Sagging skin stretches zygomaticus major fibers, resulting in deficient movement capacity	Lack of projection of the anterior nasal spine and/or premaxilla deficiency, resulting in over-contraction of depressor septi nasi and upper lip levators, which leads to gummy smile and droop of the tip of the nose Lack of bone support in chin, resulting in over-contraction of mentalis, leading to peau d’orange appearance; over-contraction of DAO leading to downturn of the oral commissure
Treatment	Inject under the muscle to increase convexity, restoring the optimal distance between the origin and insertion by enhancing the fulcrum effect; increases mechanical advantage and facilitates the action of the muscle	Inject superficial to the muscle to create a mechanical obstacle to the muscle action
Treatment effects	Increase levator muscle movement, lifting sagging structures	Reduce muscle movement Provide an obstacle to muscle excursion that causes skin deformation

*DAO* depressor anguli oris

The Case 1 patient was treated on both sides with Juvéderm Voluma^®^ (Allergan plc, Dublin, Ireland). On her right side, a bolus was injected at the level of the bone on the zygomatic arch under zygomaticus major. On her left side, two boluses were injected at the same plane (Fig. 2b). The structural support increased the muscle’s lifting action, and mechanical advantage on her left side was enhanced. Voluma was injected superficial to mentalis, depressor labii inferioris, and DAO along the labiomental angle on her left side only. This mechanical obstacle to DAO movement decreases its downward pulling effect. After treatment (Fig. 2c, right), the action of zygomaticus major is facilitated on both sides and DAO is blocked on her left, so that zygomaticus major now lifts that corner of her mouth.

**Fig. 3:**
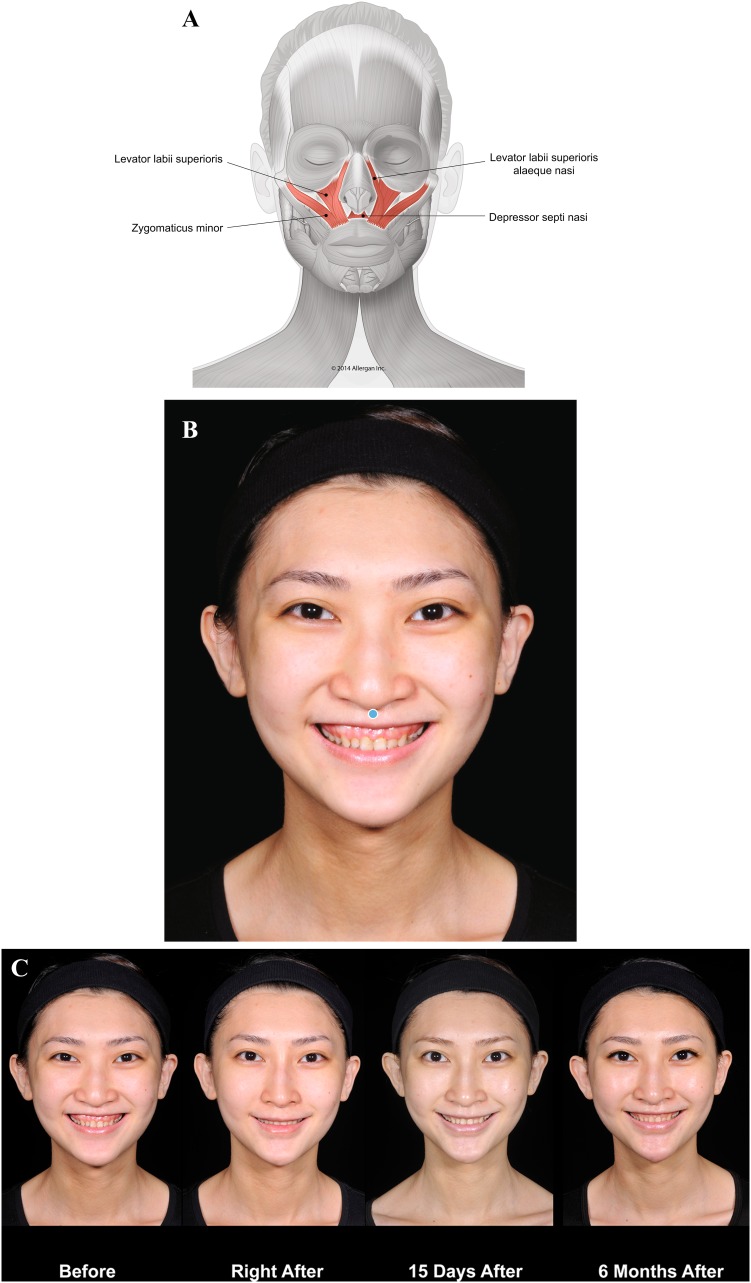
Case 2: Gummy smile. Treatment was a single bolus of Voluma (0.7 mL) injected using a 25-g cannula in the premaxilla area, at the projection of the anterior nasal spine at the level of the bone. **a** Muscles involved. **b** Injection sites (blue marking; dot = bolus injection). **c** Before treatment (left), the lack of support at the premaxilla distorts the nose position and causes a gummy smile. After treatment with Voluma (right; 6 months), the smile is more limited, correcting both the gummy smile and the distortion of the nose. This result was achieved by creating a mechanical obstacle to depressor septi nasi and upper lip levators

A lack of structural support due to bone deficiency can result in abnormal muscular contraction and surface deformation. Case 2 (Fig. 3) illustrates the contribution of a deficiency of the anterior nasal spine and premaxilla to gummy smile, and correction using injectable filler treatment. The young Asian patient presented with a lack of projection of the anterior nasal spine, retruded underdeveloped columella, and a deficit in the projection of the upper maxilla. The lack of structural and mechanical support results in excessive movement of the upper lip levators (levator labii superioris alaeque nasi [LLSAN], levator labii superioris, and zygomaticus minor), which produces gummy smile (Fig. 3c, left). The lack of support also leads to collapse of the tip of the nose and widening of the nasal flare. Voluma injected along the premaxilla and at the projection of the anterior nasal spine helped to compensate for the bone deficiency. After treatment, there is a reduction in the movement of the upper lip levators during smile and the upward retraction of the upper lip is decreased (Fig. 3c, center and right panels). Injection behind the columella has also helped to stabilize the nose.

**Fig. 4:**
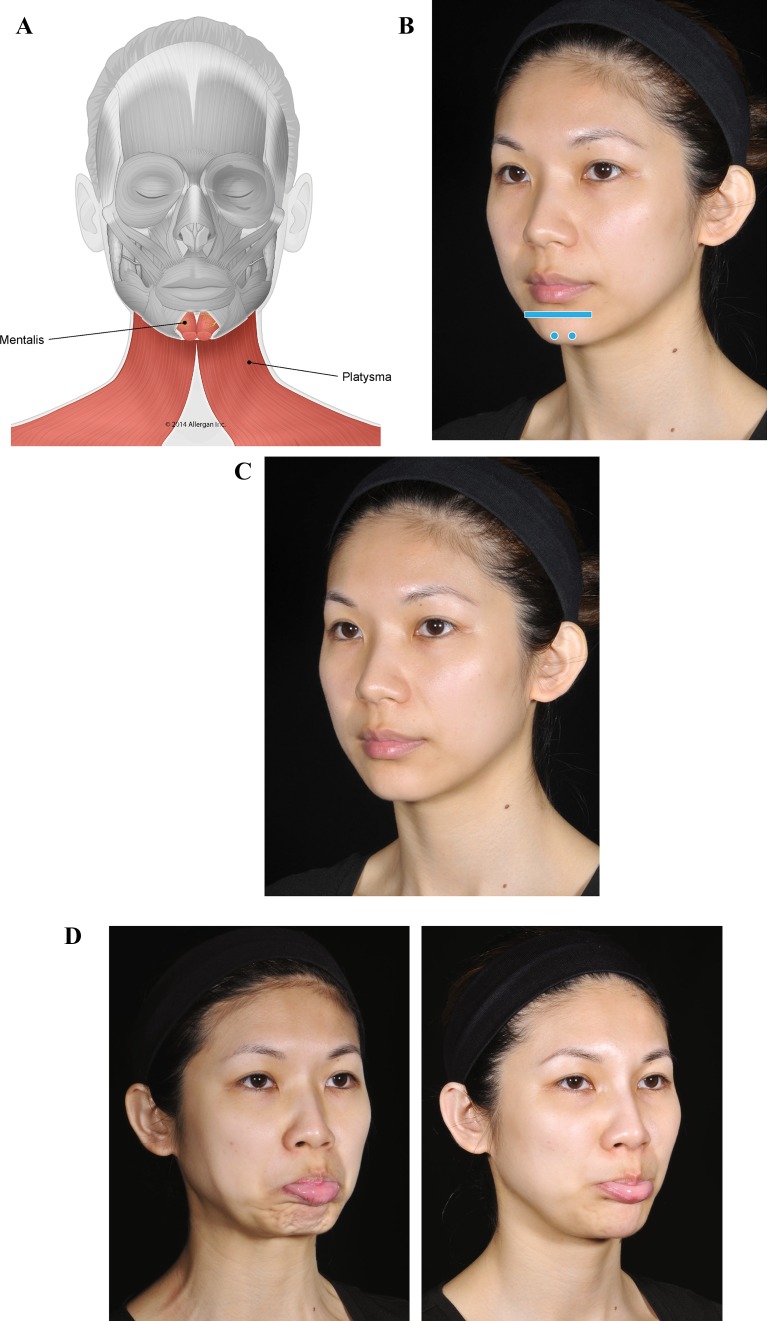
Case 3: Lack of proper bone support in the chin. The patient was treated with a total of 4 mL of Voluma (1 mL per side into the labiomental angle and 2 mL in the chin apex using a 25-g cannula). **a** Muscles involved. **b** Injection sites (blue markings; dot = bolus injection, bar = fanning). **c** Before treatment, the patient has no apparent structural deficiency at rest. **d** Pout. Before treatment (left), lack of support in the chin causes mentalis over-contraction and peau d’orange appearance. The platysma is also activated when pouting. Six months after providing a mechanical obstacle to movement with Voluma in her chin (right), the patient is able to protrude her lower lip without skin wrinkling or recruitment of platysma. Blocking over-contraction of the chin also reduces the development of hypertonic platysmal bands

The third case (Fig. 4) is a young woman who has no apparent deficiency at rest (Fig. 4c). However, on animation, a lack of proper bone support in her chin becomes evident (Fig. 4d, left). When she pouts, mentalis is activated and over-contracts. This results in upward rotation of her chin and protrusion of the lower lip, with excessive skin wrinkling and deformation. After treatment with Voluma, the patient’s pout is normal (Fig. 4d, right). The presence of a mechanical barrier at the labiomental angle and chin apex prevents the upward rotation and consequent skin wrinkling. Note that the extreme over-contraction of mentalis is blocked while preserving proper mentalis action and therefore, the patient is still able to protrude the lower lip. When over-contraction of mentalis is treated with onabotulinumtoxinA, the ability to evert the lower lip can be reduced or lost depending on the dose.

**Fig. 5:**
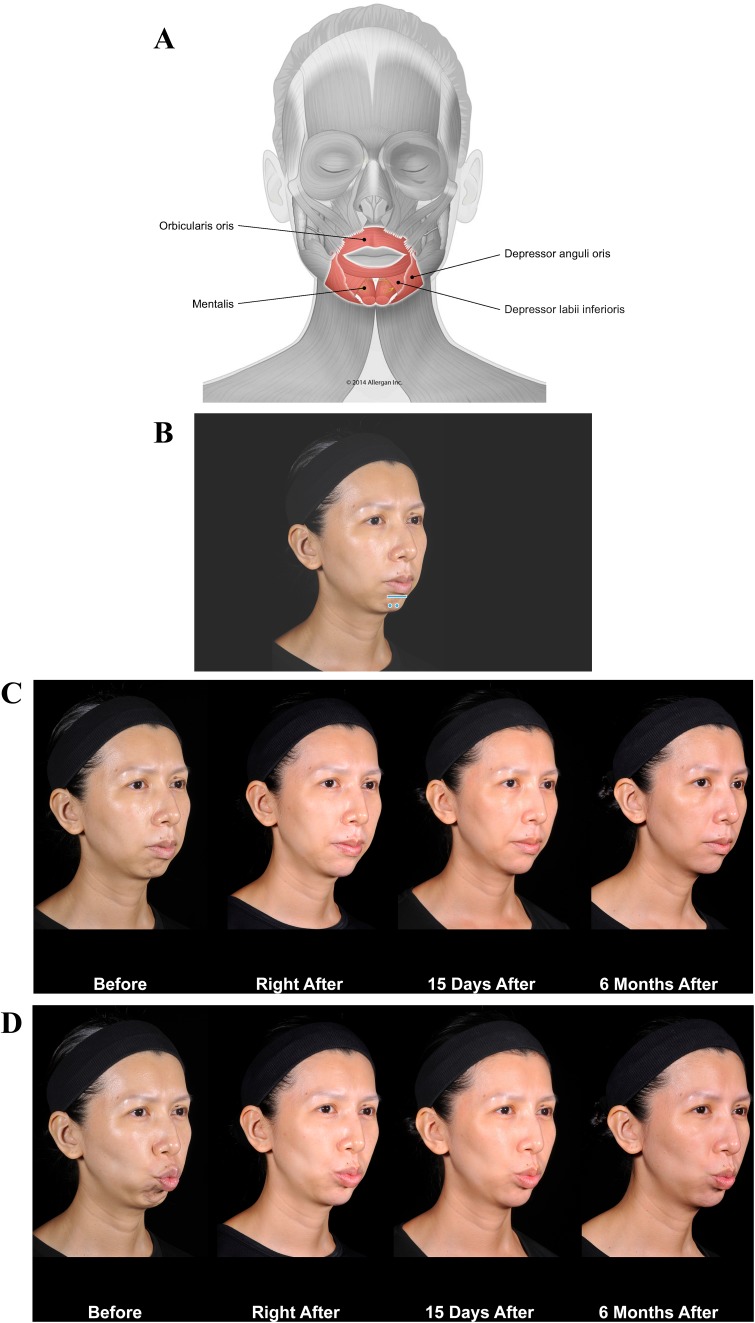
Case 4: Voluma was injected into the labiomental angle at the subcutaneous layer, superficial to depressor anguli oris and depressor labii inferioris (1.0 mL per side). Voluma was also injected at the chin apex into the deep fibers of mentalis (0.5 mL per side) using a 25-g cannula in a fanning pattern, and in a bolus pattern using a 27-g needle to reach the supraperiosteal level (0.3 mL per side). **a** Muscles involved. **b** Injection sites (blue markings; dot = bolus injection, bar = fanning). **c** At rest. Before treatment (left), wrinkling of the chin is evident at rest. Improvement in the chin is observed immediately after injection of Voluma in the labiomental angle and chin apex. **d** Purse (kiss). Before treatment (left), distortion of mentalis muscle during contraction as the patient purses her lips causes extreme wrinkling of the skin on the chin. After providing a mechanical block of mentalis muscle movement with Voluma injected in the labiomental angle and chin apex, the distortion and skin wrinkling are eliminated

In a second young woman with a lack of bone support for mentalis (Case 4; Fig. 5), distortion of the chin is evident both at rest (Fig. 5c, left) and when she purses her lips (Fig. 5d, left). Immediately after treatment, improvement in her chin is observed at rest (Fig. 5c, left center) and on animation (Fig. 5d, left center). Blocking the excessive movement of mentalis eliminates the resulting distortion and allows the patient to purse her lips with no skin wrinkling.

**Fig. 6:**
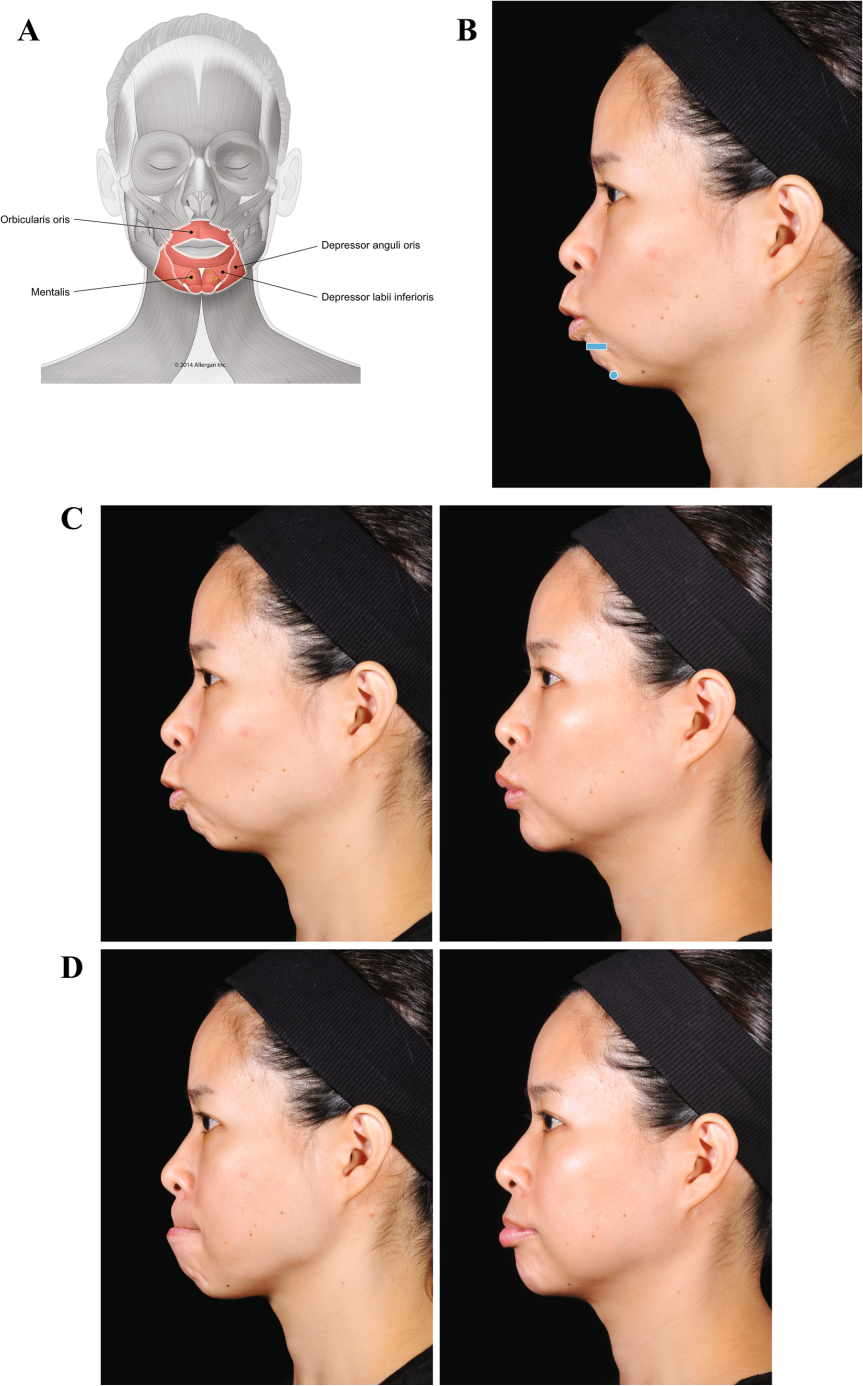
Case 5: Lack of projection at the chin. Voluma was injected into the labiomental angle, superficial to depressor anguli oris and depressor labii inferioris (0.5 mL per side), and at the chin apex into the deep fibers of mentalis (1 mL) using a 25-g cannula. Juvéderm Ultra Plus was injected in the lip border (1 mL each in cupid’s bow and lip border) using a 27-g needle. **a** Muscles involved. **b** Injection sites (blue markings; dot = bolus injection, bar = fanning). **c** Purse (kiss). Abnormal pursing movement before treatment (left) is due to a lack of support of chin and lips. Six months after treatment in the labiomental angle and chin (right), the patient is able to correctly contract orbicularis oris without deformation. **d** Pout. Filler injection in the labiomental angle and in the chin also addresses abnormal pouting movement observed before treatment (left). Six months after the injection (right), the patient is able to pout correctly

Case 5 (Fig. 6) is a young woman with notable distortion on animation (kissing, pouting). Normally, movement of the upper and lower lips during pursing is governed by nose and chin position, due to stability at the nasolabial angle and the labiomental angle, respectively. With support at these two sites, the direction of movement in a kiss is horizontal. The patient presented here lacks support at the level of the labiomental angle and chin. Instability at the labiomental angle perturbs the contraction of orbicularis oris. When she is asked to purse her lips (kiss; Fig. 6c, left), this instability causes both upper and lower lips to drop down, resulting in distortion. The normal action of mentalis in pouting leads to protrusion of the lower lip. In this case, however, the patient cannot properly protrude the lip when asked to pout (Fig. 6d, left). Instead, her lower lip everts toward the oral cavity and hides the upper lip. Treatment with Voluma in the chin and Juvéderm^®^ Ultra Plus injectable gel (Allergan plc) in the lip border allows the patient to produce a natural appearance in a kiss (Fig. 6c, right) and pout (Fig. 6d, right). With support in the soft tissue of chin and lips, mentalis and orbicularis oris contract in a more balanced, stable, and organized way. The chin and lower lip are improved by support to mentalis in the labiomental angle and chin apex. The upper lip is improved both directly by Juvéderm Ultra Plus injection and indirectly by the more organized contraction in the lower lip.

**Fig. 7:**
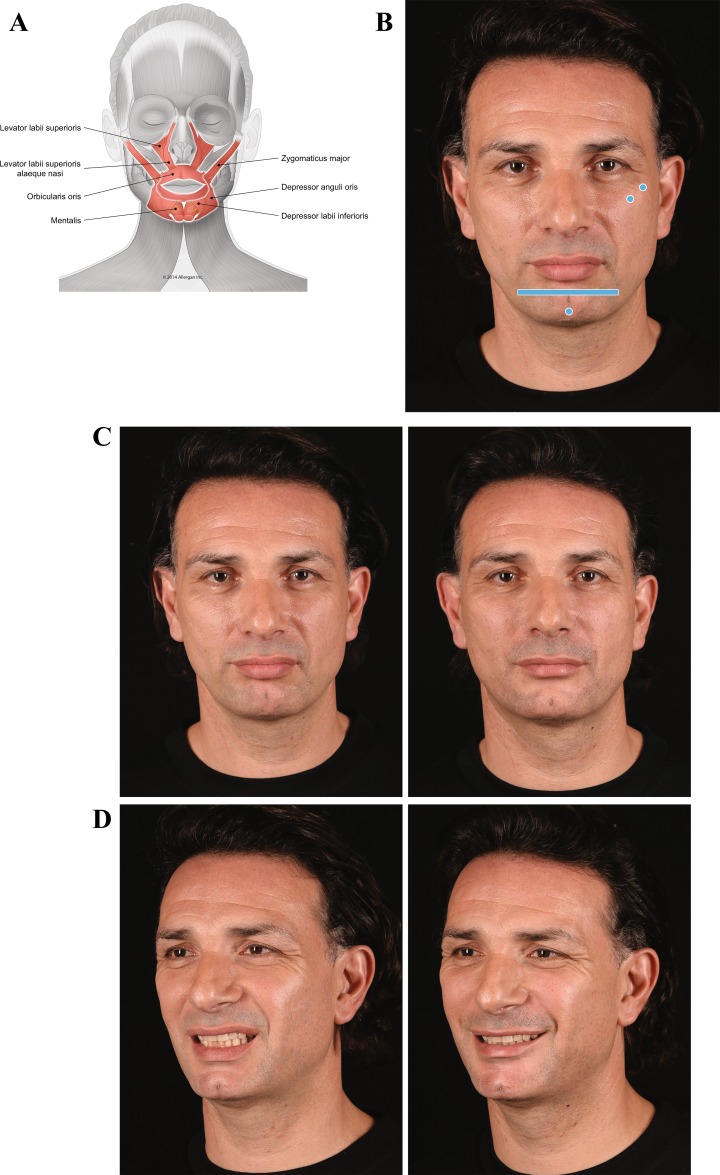
Case 6: Asymmetric smile. Voluma was injected under the zygomaticus major muscle at the level of the bone in his left cheek, 0.1 mL at the zygomatic arch and 0.2 mL at the zygomatic eminence, using a 27-g needle. Voluma was also injected superficial to the depressor anguli oris and depressor labii inferioris in the labiomental angle (1 mL) on his left and deep to the mentalis muscle at the chin apex (0.5 mL) using a 25-g cannula. **a** Muscles involved. **b** Injection sites (blue markings; dot = bolus injection, bar = fanning). **c** At rest. Before treatment (left), there is slight asymmetry at rest with a more pronounced nasolabial fold and protrusion of lower lip. Immediately after treatment (right), the nasolabial fold and the lower lip look more balanced. **d** Distortion on animation. On the patient’s left side, his nasal flare is in an upward position due to over-contraction of levator labii superioris alaeque nasi (LLSAN) before treatment (left). As a consequence, there is more upper teeth show. Depressor labii inferioris overaction leads to excessive show of lower teeth. Immediately after treatment (right), the smile line is more balanced as both LLSAN and depressor labii inferioris were blocked and zygomaticus major was facilitated. Filler treatment of lip levators and depressors controls distortion both at rest and on animation

The sixth case is a man with mild facial asymmetry, but normal facial nerve function observed at rest and on animation (Fig. 7). At rest, there is a more prominent nasolabial fold on his left side due to contraction of LLSAN, and his upper lip is slightly elevated (Fig. 7c, left). Upon animation, both LLSAN and levator labii superioris are over-contracted (Fig. 7d, left), and he presents lower teeth show on his left side when smiling. The patient was injected with two filler boluses under zygomaticus major at the level of his zygomatic arch and zygomatic eminence, respectively (Fig. 7b). He was also injected at the labiomental angle on both sides, and at the chin apex. After treatment, there is less lateral recruitment of the LLSAN and the improvement of nasolabial fold can be observed at rest (Fig. 7c, right). On animation, the upper lip is now better aligned, and as observed in his oblique view, he presents a stronger zygomatic smile due to facilitation of the zygomaticus major muscle via the fulcrum effect (Fig. 7d, right). He also presents less lower teeth show.

**Fig. 8:**
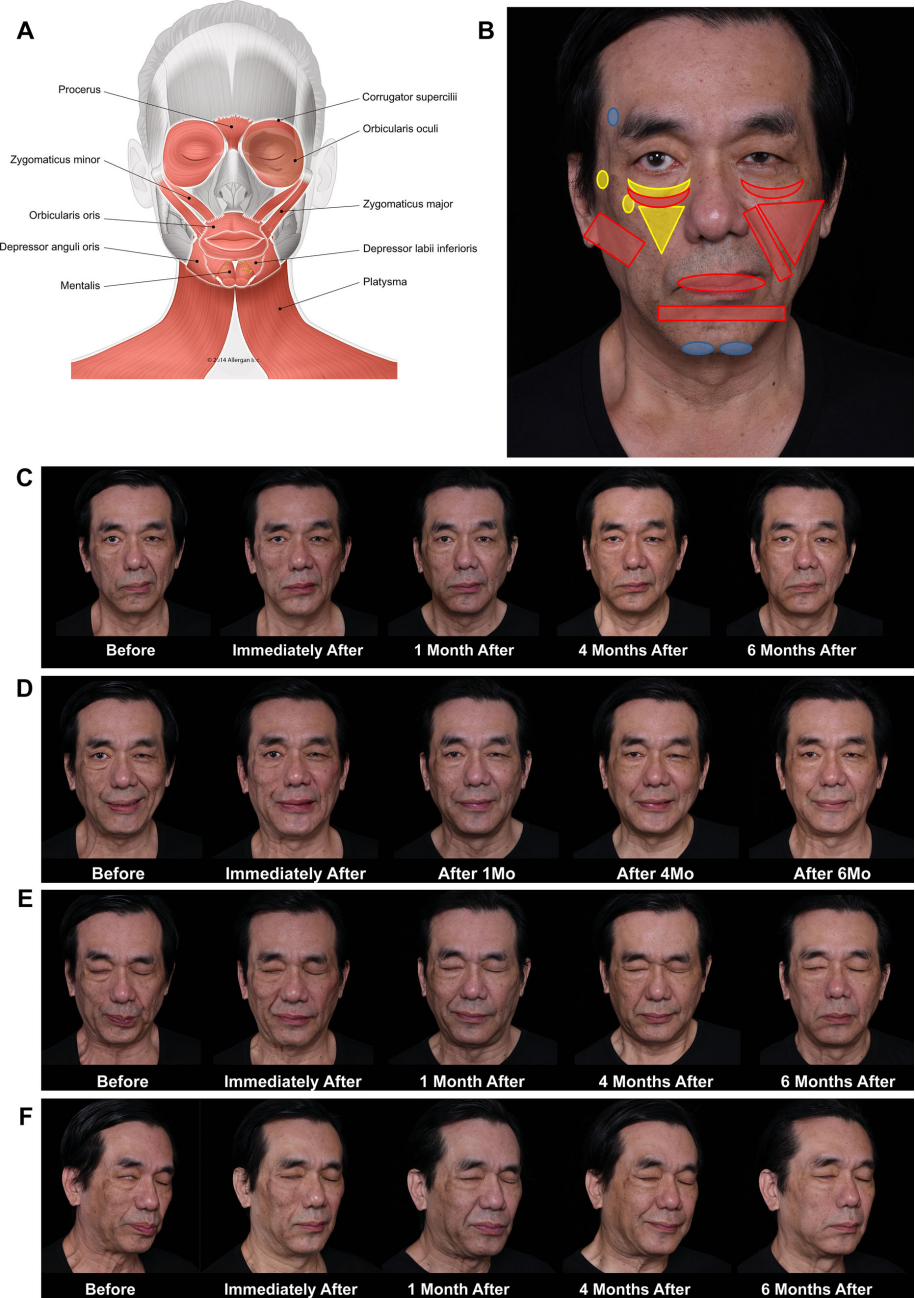
Case 7: Palsy. Treatment details are described in Table 2. No touch-up treatments were made at return visits. No botulinum toxin was used in this case. **a** Muscles involved. **b** Injection sites (yellow markings = injections under the muscle; red markings = injections superficial to the muscle; blue markings = structural injections). **c** At rest. Before treatment (left), the patient has classical signs of facial palsy on his right side. Deviation of the mouth toward his left side, with a prominent nasolabial fold and narrower left eye, is observed. After treatment, improvement of asymmetry is observed, including better eye symmetry with less scleral show on his right and better alignment of the oral commissures. **d** Smiling. Before treatment (left), there is a deviation of the oral commissure toward his left hyperkinetic side (normal side). There is also contraction of orbicularis muscle. His right side (facial palsy) presents contraction of the lateral platysmal band, indicating that the facial nerve was not completely damaged. Slight contraction of the zygomaticus major indicates there is residual activity of the zygomatic branch. Immediately after treatment, there is reduction in the upper lateral excursion of the zygomaticus major muscle on his left side. The platysma is contracting on his right side as he tries to smile. There is better positioning of the oral commissure and upper and lower lips on his right side. One to 6 months after treatment, the patient is smiling more symmetrically. His eyes are narrower bilaterally, and platysmal band contraction on his right side is apparent. **e** Closing the eyes. Before treatment (left), the patient has excessive contraction of the platysma and activation of orbicularis oris. The patient’s right eyebrow is positioned higher relative to the left. Immediately after treatment, the nasolabial fold on his right side is deeper as a result of an increased lever effect on the upper lip levators. Platysma contraction is similar, but more symmetry in the brows is observed. One month after treatment, contraction of zygomatic muscles is facilitated on his right and less recruitment of platysma is observed. Four months after treatment, the position of the eyebrows is improved and orbicularis oculi contraction appears enhanced. The patient is closing his eye with less recruitment of zygomaticus major. At 6 months, no recruitment of lateral muscles is observed. **f**. Trying to close the eyes without frowning. Before treatment (left), note the scleral show as the patient tries to close his eyes naturally. Abnormal behavior is observed in lateral muscles including orbicularis oris and platysma. Immediately after filler injection, a complete change in muscle behavior is apparent. The patient is able to close his eyes, but there is still some scleral show. No contraction of zygomaticus major muscle is observed. Four months after treatment, the zygomaticus major is even further improved. At 6 months, the patient is able to close his eyes effortlessly, without recruitment of adjacent muscles

The final case provides a most compelling demonstration of the use of injectable fillers for myomodulation. Case 7 (Fig. 8) is a middle-aged man with facial palsy on the right side resulting from acoustic neuroma surgery in 2011. Prior to injectable filler injection, no treatment to improve the facial asymmetry was performed. Before treatment (Fig. 8c, left), the patient had classic signs of facial palsy on his right side: a lack of function of orbicularis oculi and zygomaticus major, with scleral show and skin laxity. A more prominent nasolabial fold and lip deviation toward his left side is observed. The patient’s smile (Fig. 8d, left) shows even greater deviation of the oral commissure toward his hyperkinetic side (left). On his facial palsy side (right), the contraction of lateral platysmal band shows that the facial nerve is not completely damaged. Slight contraction of the zygomaticus major, indicated by the presence of a dynamic line at the modiolus level and worsening of the skin excess at the lower eyelid, suggests that there is residual activity in the zygomatic branch. When the patient tries to close his eyes tightly (Fig. 8e, left), abnormal coordination of facial muscles is demonstrated by his frown, excessive contraction of platysma, and activation of orbicularis oris on his left with the eyebrow elevated. When he tries to close his eyes without frowning (Fig. 8f, left), scleral show and abnormal behavior of lateral muscles are observed.

Mimetic muscles on both sides were treated with filler injections (Fig. 8b), with depth of injection the major difference between the two sides. The treatment of the patient’s right side focused on lifting the cheek and stabilizing the lips. Filler was injected deep to muscle fibers, at the level of the bone wherever possible, to create a lever effect to increase muscle movement. On his left (hyperkinetic) side, fillers were injected superficial to the muscle, at the subcutaneous level, in order to reduce contraction by providing a mechanical obstacle to muscle contraction. Filler products and volumes used in this case are shown by injection site in Table 2. Topographical muscle anatomy and mimetic animation assessment during the injection were used to guide the treatment. There was no need for the use of electromyography.

**Table 2:** Patient treatment record, Case 7

Injection sites	Product	Technique	Volume (mL)	
			Right side of face	Left side of face
Temple	Voluma	Structural, supraperiosteal; needle	1.0	–
Tear trough and palpebral malar sulcus	Volbella	Right: Microaliquots, *superficial to* orbicularis oculi; cannula *Bilateral*: Microaliquots, *deep to* orbicularis oculi; cannula	1.5	1.0
Zygomatic arch and eminence of zygomatic bone	Voluma	Right: Bolus, supraperiosteal, *deep to* zygomaticus major and minor; needle Left: Fanning needle subcutaneous *superficial to* zygomaticus major and minor	1.25	0.8
Anteromedial cheek	Voluma	Right side: Fanning, *deep to* upper lip levators; cannula Left: Fanning, subcutaneous, *superficial to* the upper lip levators; cannula	1.25	0.3
Lower lateral cheek	Voluma	Skin lifting for structural support: Fanning cannula subcutaneous, *superficial to* SMAS	1.0	1.0
Nasolabial fold	Voluma	Left: Linear, subcutaneous, *superficial to* the upper lip levators; cannula	–	1.8
Lip	Juvéderm Ultra Plus	Linear, vermillion; cannula Linear, lip border; needle	0.75	0.25
Labial mental angle	Voluma	Structural support to lower lip: Linear, subcutaneous, *superficial to* lower lip depressors; cannula	1.0	1.0
Chin	Voluma	Structural, supraperiosteal; needle	0.9	0.9
Total			8.65	7.05

Injection sites are shown in Fig. 8b

*SMAS* superficial musculoaponeurotic system

Immediately after the injections, better symmetry and muscle coordination were observed. On smiling, there is a reduction in the upper lateral excursion of the zygomatic major muscle on his left side and better positioning of the oral commissure and upper and lower lips (Fig. 8d, immediately after). When closing his eyes (with effort) immediately after treatment, a deeper nasolabial fold is apparent on his left, indicating a change in muscle behavior between his right and his left side (Fig. 8e, immediately after). When he tries to close his eyes without effort, there is improvement in scleral show immediately after treatment (Fig. 8f, immediately after).

Over the 6 months following treatment, the patient was instructed to practice closing his eyes and smiling more symmetrically using a mirror. Although improvement resulting directly from filler treatment is evident in the patient’s smile and eye closure in the photographs taken immediately after treatment (Fig. 8d–f), the symmetry of his smile continues to improve progressively with exercise (Fig. 8d, 1–6 months). One month after treatment, contraction of zygomatic muscles on his right side appears enhanced and there is less recruitment of platysma (Fig. 8d, 1 month). By 4 months after treatment, contraction of orbicularis oculi and position of the eyebrows are improved (Fig. 8e, 4 months), the patient closes his eye more naturally, and enhanced zygomatic action is observed (Fig. 8f, 4 months). At 6 months, he closes his eyes effortlessly without recruitment of adjacent muscles (Fig. 8e and f, 6 months).

## Summary and Conclusions

Deficits in facial structure can yield abnormal muscle action reflected at the skin and across the face. When structural support is absent or lost, muscle action is altered, affecting the balance in activity between muscles. Examining the interactions between facial structure and muscle movement—and recognizing unbalanced action in muscle synergists and antagonists—allows the clinician to understand the effects on appearance both at rest and on animation. The cases presented here provide support for the use of myomodulation, addressing muscle movement with injectable fillers in the treatment of facial structural deficiencies. Injectable filler treatment can be used to support muscle movement or block overaction regardless of whether the imbalance is due to a structural deficiency or a loss of volume in aging, and indeed, the clinician does not need to isolate the contribution of these factors to an imbalance in order to use fillers to treat it.

The injection details outlined in the cases presented here are provided as an overview of how myomodulation with fillers can be used, and not as a guide to treatment. Further investigation examining specific uses for this approach will be needed in order to present techniques for the administration of injectable fillers for treating particular deficiencies. In addition, future studies using electromyography to measure changes in muscle activity before and after treatment, together with videography to closely compare appearance on animation, will be needed to confirm hypotheses about changes in muscle activity underlying observed esthetic effects and improvement in palsy patients.

The framework presented here is based on experience treating patients with facial palsy, structural deficits, and effects of aging. The cases included are young and middle-aged individuals treated for effects that likely result, at least in part, from structural deficiencies. Based on an understanding of the importance of structure and stability in muscle function, experience with cases such as these suggests that where structure is lacking, myomodulation with fillers should be considered before neurotoxin injection. Filler treatment can be used to correct structural deficiency, support muscles to facilitate their action, and provide an obstacle to extreme muscle excursion and depressor contraction. Neurotoxins, to be sure, are a powerful tool of primary importance for modulating facial muscle activity. However, injectable fillers have a unique and important role to play, in that they can both support weak muscle action and locally block overacting muscles. When used together with fillers, toxins may perform more effectively in blocking over-contracting depressors and fine-tuning the balance of activity among synergist and antagonist muscle groups. Using these tools together, the clinician can reestablish natural structural conditions and rebalance muscle movement to restore facial appearance to that of a typical youthful individual.
